# Evaluation of crude larval protein and recombinant somatic protein 26/23 (rHcp26/23) immunization against *Haemonchus* *contortus* in sheep

**DOI:** 10.14202/vetworld.2017.758-763

**Published:** 2017-07-08

**Authors:** Omnia M. Kandil, Khaled A. Abdelrahman, Hatem A. Shalaby, Seham H. M. Hendawy, Nadia M. T. Abu El Ezz, Somia A. Nassar, James E. Miller

**Affiliations:** 1 Department of Parasitology and Animal Diseases, National Research Centre, Dokki, P.O. Box 12622, Giza, Egypt; 2 Department of Pathobiological Sciences, School of Veterinary Medicine, Louisiana State University, Baton Rouge, LA 70803, USA

**Keywords:** antigen, *Haemonchus contortus*, immunization, larval, rHcp26/23, sheep

## Abstract

**Aim:**

The aim of this study was to evaluate the potential possibility of crude larval and recombinant (rHcp26/23) antigens of *Haemonchus contortus* for immunization to control sheep hemonchosis.

**Materials and Methods:**

A total of 21 lambs were divided into five groups. Lambs were immunized with larval and recombinant (rHcp26/23) proteins at day 0 and day 14 and after that challenged with 5000 infective larvae of *H. contortus* on day 42. An unvaccinated positive control group was challenged with L3 in the meantime. An unvaccinated negative control group was not challenged.

**Results:**

Fecal egg count reduction taking after challenge for rHcp26/23 and larval antigens was 92.2% and 38.2%, respectively, compared with the positive control group. Vaccine incited protection in rHcp26/23 and larval immunization was reflected in significant (p<0.05) decreases in worm burden; 59.9% and 40.1%, respectively.

**Conclusion:**

Recombinant rHcp26/23 vaccine induced a partial immune response and had immune-protective effect against sheep hemonchosis.

## Introduction

Lambs are infected by an assortment of gastrointestinal nematodes. Their effects range from slight to lethal, depending on numerous elements, for example, the site and degree of infection, method of feeding of the nematode, and the nourishing and physiological status of the host [1,2]. *Haemonchus contortus* feeds on blood obtained by damaging the abomasal mucosa resulting in mild anemia to mortality, especially in younger animals [3].

The control of nematode parasite is dependent on chemical antihelminthic treatment and pastures management. Moreover, parasite strains resistant to these antihelminthic drugs had been on the increase [4-6]. Today, vaccination control measures are viewed as the most ideal instruments [7,8]. Numerous studies concentrated on the identification and characterization of immunogenic antigens of *H. contortus*, and their capability to incite protective immunity by immunization. Among the most encouraging antigens, crude, larval, and the somatic antigen p26/23 are usually utilized [2,7-10]. Results from vaccination trials have differed in protection levels from 32.2% to 90% decrease in parasite egg shedding and 61-78% reductions in worm burden in the abomasa of immunized sheep [7,11,12]. The protein fraction p26/23 from adult worms was immunoprotective in lambs challenged with *H. contortus* [12]. As of late, the substance of this fraction protein has been analyzed, and the real protein present has been purified, immunolocalized, and mostly sequenced, cloned and expressed [13,14]. What’s more, the two antigens H-gal-GP and H11 isolated from intestinal cells of *H. contortus* have continually protection to a degree which would surpass antihelmintic treatment administration (i.e., >80% adequacy in >80% of the herd; and which would in this way be financially helpful. Native H-gal-GP and H11 have each been appeared to diminish fecal egg counts (FEC) by more than 90% in immunized sheep and, when utilized as a part of mix; their impact in a controlled field trial was very viable for grazing Merino sheep [15].

In this study, two unique antigens crude larval antigen and recombinant protein (rHcp26/23) were prepared and after that characterized by immunoblot. The goal of this study was to compare the immune response evoked by vaccination with the prepared antigens.

## Materials and Methods

### Ethical approval

This study was approved by Medical Research Ethics Committee (National Research Centre, Egypt) under registration number (16050). The experiments were conducted in accordance with the guidelines laid down by the International Animal Ethics Committee and in accordance with local laws and regulations.

### Sample collection

*H. contortus* adult worms were obtained from abomasa of slaughtered sheep at different abattoirs in Egypt. L3 were obtained from cultured eggs from female worms according to Soulsby [16]. Identification of the collected worms and L3 was done according to Whitlock [17]. The collected L3 was washed with phosphate-buffered saline (PBS) and stored at 4°C for infection purposes and challenge trials.

### Crude L3 antigen of H. contortus

Balady lambs were housed in a hygienic isolated pen and fed a balanced ration, offered fresh water, parasitologically examined for eggs per gram (EPG) to ensure that it was free from any helminthes, and kept under observation for 30 days to acclimate before the experiment. Two balady lambs 2-3 months of age were experimentally infected with 5000 L3. Eggs were obtained from infected sheep after 21 days of infection. L3 were obtained from fecal culture and then baermannization. Preparation of antigen was done according to Alunda *et al*. [18]. In brief, 100 g of feces was weighed and incubated for 2-3 weeks at room temperature. During this time, it was regularly checked for desiccation, moistened if necessary, and ventilated for 1 h/day. After incubation, L3 was harvested by baermanization for 24 h. The sediment containing the accumulated L3 larvae was obtained. The total protein content was estimated by Lowry protein assay to determine the total level of protein in the antigen according to Lowry *et al*. [19], and the L3 antigen was analyzed by sodium dodecyl sulfate polyacrylamide gel electrophoresis (SDS-PAGE) and western blotting (WB) using pooled sera from experimentally infected positive control lambs according to Laemmli [20] and Towbin *et al*. [21]. Concisely, L3 antigen was resolved using 10% polyacrylamide gel under reducing conditions. After electrophoresis, one gel was stained with Coomassie brilliant blue R-250 dye, and the other was transferred to 0.45 nitrocellulose membrane and blocked for 1 h in 1% dry skimmed milk dissolved in PBS pH 7.2, then probed overnight against experimentally infected positive and negative control lamb sera at 1:100 in tris-buffered saline (TBS) with 0.5% bovine serum albumin (BSA). The nitrocellulose strips were incubated with anti-sheep immunoglobulin G (IgG) (whole molecule) peroxidase antibody produced in donkey (Sigma-Aldrich, USA) in 0.5% BSA/TBS buffer for 1 h at dilution 1:1000. The reactive bands were developed by incubation of the blot in the substrate solution (1-chloronaphthol [Sigma-Aldrich, USA], one tablet [30 mg/1 ml methanol] added to 10 ml methanol, 39 ml TBS, and 30 µl 30% H_2_O_2_) for 5 min.

### Recombinant somatic H. contortus protein 26/23 (rHcp26/23)

Adult male *H. contortus* was used in an RNA extraction kit protocol (Qiagen, Germany) according to Garcıa-Coiradas *et al*. [14]. Reverse transcriptase polymerase chain reaction (PCR) was carried out in two independent steps: Synthesis of cDNA (1^st^ strand cDNA Synthesis Kit, AMV, Roche) using hexanucleotide random primers with BamHI and HindIII restriction targets:

F BamHI (5\GGATCCGCAGGACTGTTCGC ACAT3\) and R HindIII (5\AAGCTTTCAGTCTTT CGCGGACTTG3\).

The PCR amplification included denaturalization for 3 min at 94°C, followed by 30 cycles (95°C, 1 min) with one annealing elongation step at 71°C for 1 min and, finally, an elongation step at 72°C for 10 min. PCR was carried out in a PTC-100 (MJ Research Inc.). The resulting fragment was cloned in the vector pGEM-T (Promega), and the construct was used to transform *Escherichia coli* XL2-blue. Positive bacterial colonies were identified by PCR employing the primers; SP6 (5\ATTTAGGTGACACTATAGAA3) and T7 (5\TAATACGACTCACTATAGGG3\). Minipreps were prepared with PCR-positive colonies (QIAprep Spin Miniprep Kit Qiagen).

The insert was cloned in the expression vector pQE30 (QIAexpress Vector, Qiagen), and the construct was employed to transform *E. coli* M15 (Qiagen). Positive bacterial colonies were identified by PCR employing the primers as follows: for the plasmid FpQE (5\GAATTCATTAAAGAGGAGAAA3\), for the insert R (5\TCAGTCTTTCGCGGACTTG3\). The nucleotide sequence of PCR products and the positive bacterial clones in *E. coli* XL2-blue were determined by the Animal Health Research Institute. The expression of the recombinant protein (rHcp26/23) was carried out with a PCR positive clone of *E. coli* cultured in Luria broth medium. Cell pellets from cultures were resuspended, and protein was solubilized in both denaturing and nondenaturing conditions. In the purification under denaturing condition, the recombinant His6tagged p26/23 was purified in 10 cm × 1 cm columns (BioRad) of Ni-NTA agarose (Qiagen). Purification of the recombinant protein was carried out and was analyzed by SDS-PAGE and WB [20,21] using pooled sera from vaccinated lambs with the fraction p26/23. The protein markers used were protein marker M1: Genscript, Cat. No. M00505 and protein marker M2: Genscript, Cat. No. MM0908.

### Vaccine protocol

The formulated crude larval and recombinant *H. contortus* vaccines were safety tested in rabbits before vaccination trails [22], and shown to be safe. Twenty one, 3-month-old helminthes-free lambs were obtained locally and housed in an isolation facility. The animals did not graze with their mothers, and FEC was performed quantitatively using a McMaster technique [16] to ensure that it was helminthes-free. Lambs were distributed in a stratified manner (by live weight) onto 5 experimental groups. Group 1 (n=5), immunized with rHcp26/23; Group 2 (n=5), immunized with crude L3 antigen; Group 3 (n=5), received only adjuvant, challenged control; Group 4 (n=3), challenged only, positive control; Group 5 (n=3), unvaccinated and unchallenged negative control. Lambs from Groups 1 and 2 received immunizing injections (intramuscular and subcutaneous in the inner thigh and hind legs) on days 0 and 14. The first injection (100 µg protein) was administered in 1 mL Freund’s complete adjuvant (Sigma-Aldrich, USA) and the second injection was administered in 1 mL Freund’s incomplete adjuvant (Sigma-Aldrich, USA). On day 42, Groups 1-4 were challenged with 5000 *H. contortus* L3 [9]. Sera from different animals’ group were weekly collected from 0 days till end of the experiment to evaluate the sero-conversion of the animals.

### Evaluation of vaccines

#### FEC and worm burden

FEC was performed by the McMaster technique [16] at 2 days intervals from 17 days after challenge infection until the end of the study. Sheep were euthanized and slaughtered humanely at 50 days after challenge for abomasal worm count determination. Abomasa were immediately removed, opened and the contents collected in a container. The empty abomasa were washed thoroughly with warm 0.85% NaCl solution to remove adhering worms and subsequently soaked thoroughly, and the washing was sampled. Worm counts were made on 1/50 aliquots on both washing and abomasal content.

### Immunological assay

Humoral immune response (estimation of *H. contortus* serum antibody level using enzyme-linked immunosorbent assay [ELISA] technique) was done according to Kandil *et al*. [23]. Each prepared antigen was used to test its respective vaccinated group with the positive and negative control groups from zero days to the end of the experiment. Briefly, the wells were coated with 100 µl of each diluted antigens; L3 and rHcp26/23 at the concentration of 0.2 µg/well in carbonate-bicarbonate buffer, pH 9.6 and incubated for 1 h at 37°C then incubated overnight at 4°C. After blocking, 100 µl/well of diluted serum at 1:200 was added as duplicate, and the plates were incubated for 1.5 h at 37°C. Then, 100 µl/well of conjugate; anti-sheep IgG (whole molecule) peroxidase antibody produced in donkey (Sigma-Aldrich, USA) diluted at 1:1000 in diluting buffer was added and incubated for 1 h at 37°C. The plates were washed extensively with washing buffer. 100 µl/well of substrate solution (20 mg of O, phenylenediamine [Alfa Aesar, UK] was dissolved in 50 ml substrate buffer, pH 5 and 25 µl 30% H_2_O_2_) was added to all wells and the plates were incubated 10 min at 37°C. The optimum color development was stopped by addition of 100 µl of stopping buffer (5% SDS) to each well. OD was read at wavelength of 450 nm with an ELISA reader (Bio-Tek, Inc., ELx, 800 UV). The sera were considered to be positive when the absorbance values were more than the cutoff value. The cutoff value was calculated as mean value plus 3 times the standard deviation of optical density value of negative control sera.

### Statistical analysis

Data were statistically analyzed by ANOVA that was used to test for differences between the immunized and control means, and Duncan’s test was used to separate means at stated level (p<0.05) using SPSS computer program.

## Results

### Evaluation of the crude L3 and recombinant somatic protein rHcp26/23 H. contortus antigens

**Figure-1:**
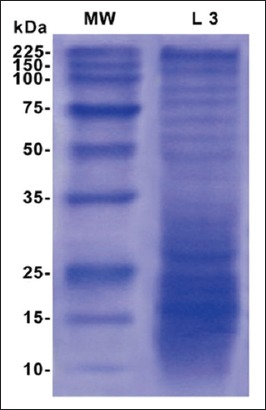
Sodium dodecyl sulfate polyacrylamide gel electrophoresis of crude L3 antigen of *Haemonchus contortus*. MW: Unstained broad range protein marker, Promega. L3: Crude L3 antigen of *H*. *contortus*.

**Figure-2:**
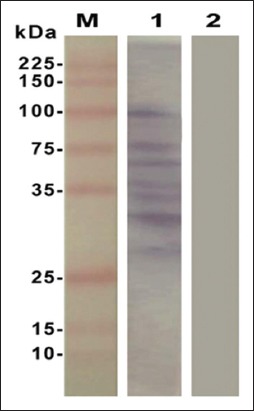
Immunoblotting analysis of crude L3 antigen of *Haemonchus contortus*. M: Unstained broad range protein marker, Promega. 1: Pooled sera from experimentally infected positive sheep against crude L3 antigen of *H. contortus*. 2: Negative sheep serum.

Electrophoretic profile of the L3 antigen showed multiple fractions in both high and low molecular weights (Figure-1). L3 antigen gave 13 protein bands with different molecular weights (187, 112, 88, 76, 66, 53, 45, 32, 28, 21, 17, 14, and 10 kDa). The immune-blot reaction showed that 7 (100, 75, 66, 35, 34, 32, and 28 kDa) antigenic bands of crude L3 antigen were recognized using positive sera (Figure-2).

**Figure-3:**
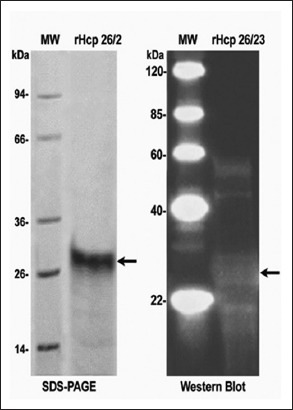
Sodium dodecyl sulfate polyacrylamide gel electrophoresis (SDS-PAGE) and Western blot analysis of recombinant somatic *Haemonchus contortus* protein 26/23 (rHcp26/23). MW of SDS-PAGE: Genscript, Cat. No. M00505. rHcp26/23: Recombinant somatic *H. contortus* protein 26/23 antigen. MW of WB: Genscript, Cat. No. MM0908. rHcp26/23: Recombinant somatic *H. contortus* protein 26/23 antigen against pooled sera from vaccinated lambs with the fraction rHcp26/23.

The electrophoretic and blotting analysis revealed that the rHcp26/23 protein was identified at 26 kDa using anti-His antibody (Figure-3).

### Immunological assay

**Figure-4:**
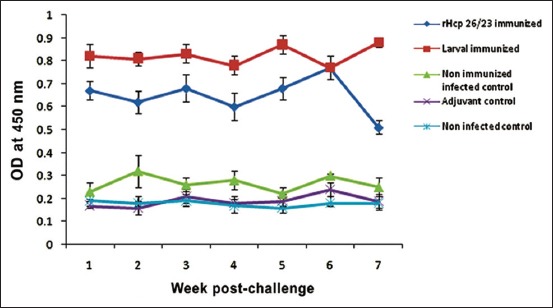
Appearance of anti-*Haemonchus* antibodies in sera of recombinant protein rHcp26/23 and L3 immunized sheep compared to control groups at different intervals postchallenge.

Antibody level achieved most elevated values for rHcp26/23 and L3 on weeks 6 and 5, respectively, and stayed high until the end of the study. On contrary, antibodies levels in sera of adjuvant, non-immunized infected, and non-infected controls were low (Figure-4).

### FEC and worm burden

**Figure-5:**
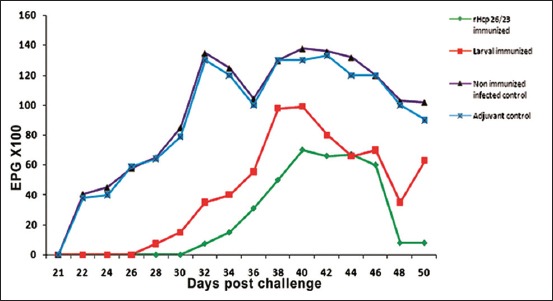
Mean fecal egg count (eggs per gram of feces) for recombinant protein rHcp26/23 and L3 immunized sheep compared to control groups at different intervals postchallenge.

In adjuvant and non-immunized infected controls, the first eggs were detected on day 22 after L3 challenge. The counts increased gradually during the sampling period, reaching peak levels at 40 days post-challenge (Figure-5). Whereas in rHcp26/23 and L3 immunized groups, positive egg counts were observed from day 32 to 28, respectively. From this day onward, the FEC was lower in the immunized sheep than in the non-immunized sheep. FEC ranged from 750 to 7000 EPG for the sheep immunized with rHcp26/23 and 750-9900 EPG for those immunized with L3 antigen. FEC in non-immunized infected controls ranged from 3800 to 13800 EPG. At day 50, mean EPG was reduced in the groups immunized with rHcp26/23 (800±72) and L3 (6300±504) contrasted with the control (10,200±816). Accordingly, the mean FEC for immunized groups was reduced by 92.1% with rHcp26/23 and 38.2% with L3 antigen, at day 50. This study revealed that there was a significant (p<0.05) reduction in mean FEC for rHcp26/23 immunized group compared to the control group.

Vaccine incited protection in rHcp26/23 and L3 immunized groups was reflected in significant reductions in worm burden; 59.9% (p<0.05) and 40.1% (p<0.05), respectively, contrasted with the non-vaccinated infected control group. Less worms were recovered from the group vaccinated with rHcp26/23 (297.7±32.8) contrasted with the control (742.8±66.9) or the L3 immunized group (445.3±57.9).

## Discussion

In this study, the prospective efficacy of recombinant rHcp26/23 of *H. contortus* and its crude L3 antigens to protect against homologous infection was investigated in sheep. The results proved that sheep was partially protected with reductions in FEC and abomasal worm burden for rHcp26/23 immunized group (92.1% and 59.9%, respectively) and minor reductions were achieved with L3 antigen (38.2% and 40.1%, respectively). The immunized groups recorded higher anti-*Haemonchus* antibodies levels after challenge infection compared with their non-immunized groups. Results demonstrated that little amounts of two somatic peptides (rHcp26/23) inspired in partially protective response against *H. contortus* challenge in sheep. The level of protection accomplished with rHcp26/23 was similar to that reported by Jasmer and McGuire [24] utilizing gut protein from *H. contortus* in goat and those accomplished in sheep utilizing excretory-secretory proteins [25], purified antigen from larvae [7], or diverse adult membrane antigen [24]. Jasmer and McGuire [24] showed that gut antigens communicated in adult *H. contortus* were available in L3 might be imperative. Experiments described here were not designed to test whether pre-adult parasite stages were affected by immunity to adult gut antigens. Nonetheless, results suggest this is conceivable since both third- and fourth-stage larvae are tissue dwelling. The presentation of protective gut antigens expressed in these tissue stages could stimulate good immune responses in vaccinated animals when they are exposed to natural challenge [26]. Therefore, selecting specific antigens expressed in the adult gut and larvae may be an important strategy in vaccine development. Likewise, Coyne and Brake [27] revealed the potential possibility of propagating parasite-derived cell populations in an *in vitro* tissue culture environment in a way that holds their capacity to express immunoprotective antigenic fractions. Understanding of experimental findings cleared that sheep with the best antigen-specific humoral immune responses (IgG titre 1/3125) also showed a level of lessened abomasal *H. contortus* hatchlings loads (60% decrease) [27]. The rates of egg and worm diminishments were additionally equivalent to those got by Piedrafita *et al*. [7], Fawzi *et al*. [8], and Fawzi *et al*. [12] utilizing the same low molecular weight peptides from *H. contortus* in sheep. On contrary, Garcıa-Coiradas *et al*. [14] reported that regardless of the strong immune response elicited by the immunization of lambs with the recombinant protein (rHcp26/23) no protection against the *H. contortus* challenge was found. They recommended numerous explanations behind the lacking denaturalization of the recombinant proteins and glycosylation of the defensive antigens. Whereas, in this study, complete purification of the expressed protein was done since carbohydrate moieties might mask the potential protective responses [28].

In this study, the decrease of aggregate worms and FEC in both vaccinated groups demonstrated an alternate pattern with the ELISA optical densities of sera. This may be attributed to the poor antigenicity of somatic antigen contrasted with larval and different antigens [23]. Vaccination did not totally kill nematodes from immunized animals but rather it could be proficient to lessen field contamination and thus the level of reinfection. The greater value of vaccines might be in diminishing field pollution than killing the nematodes in the host [12].

Finally, vaccinations are essential part of a group well-being administration program. The vaccination program ought to kill worms at the top of infection and avoid reinfection of field during high-risk periods. Two vaccinations are prescribed, from the author’s feeling, toward the start of the dry season and two vaccinations toward the start of the rainy season. The interim between the first and second vaccinations ought to be 2-3 weeks. The vaccination toward the start of the dry season is done to dispense with current parasite burden, empowering lambs to better adapt to the dietary stress during the dry season. A vaccination before the rainy season will forestall contamination of fields at a time when conditions are getting to be positive for egg and larval development.

## Conclusion

The potential of recombinant protein rHcp26/23 of *H. contortus* and crude L3 protein to protect against homologous infection was investigated in sheep. The findings suggested that sheep was partially protected with reductions in FEC and abomasal worm burden for rHcp26/23 immunized sheep with lower reductions achieved with L3 antigens.

## Authors’ Contributions

OMK and JEM designed the plan of work. OMK supervised and provided guidance for the research work. OMK, KAA, HAS, SHMH, NMTA, and SAN carried out sample collection, sample processing, conducted the experiment and the laboratory work of the samples. OMK, KAA, HAS, and SHMH analyzed and discussed the resultant data. OMK, KAA, and HAS preparing the manuscript. OMK and SHMH revised and reviewed the manuscript for publication. All authors read and approved the final manuscript.
